# Efficacy and safety of switching from bosentan or ambrisentan to macitentan in pulmonary arterial hypertension: A systematic review and meta-analysis

**DOI:** 10.3389/fcvm.2022.977110

**Published:** 2022-12-07

**Authors:** Jie Li, Zu-Yuan Yang, Shang Wang, Ping Yuan, Qin-Hua Zhao, Su-Gang Gong, Hong-Ling Qiu, Ci-Jun Luo, Hui-Ting Li, Rui Zhang, Wen-Hui Wu, Jin-Ming Liu, Lan Wang, Shan-Shan Liu, Rong Jiang

**Affiliations:** ^1^Department of School of Medicine, Tongji University, Shanghai, China; ^2^Department of Cardio-Pulmonary Circulation, Shanghai Pulmonary Hospital, School of Medicine, Tongji University, Shanghai, China; ^3^Department of Hematology, The Affiliated Hospital of Qingdao University, Qingdao, China

**Keywords:** endothelial receptor antagonists, pulmonary arterial hypertension, macitentan, 6-min walk distance, bosentan, ambrisentan

## Abstract

**Background:**

There is little evidence of the effectiveness of switching from the endothelin receptor antagonists (ERAs) bosentan and ambrisentan to a novel ERA, macitentan, in patients with pulmonary arterial hypertension (PAH). Therefore, a systematic review and meta-analysis was performed to evaluate the efficacy and safety of patients with PAH switching from other ERAs to macitentan.

**Methods:**

We retrieved the relevant literature published before January 2022 for the meta-analysis from the PubMed, EMBASE, and Cochrane Library databases. Efficacy included changes in the 6-min walk distance (6MWD), World Health Organization functional class (WHO-FC), N-terminal pro-brain natriuretic peptide (NT-proBNP) levels, hemodynamics, echocardiography and survival.

**Results:**

Nine studies, consisting of 408 PAH patients, that met the inclusion criteria were included. The switch from bosentan or ambrisentan to macitentan effectively increased the 6MWD by 20.71 m (95% CI: 10.35-31.07, *P* < 0.00001, *I*^2^ = 0%). Six months after conversion, the tricuspid annular plane systolic excursion was found to improve from 19.0 ± 4.0 to 21.0 ± 5.0 mm in adults and from 16.00 ± 5.0 to 18.25 ± 4.8 mm in children. Ordinal logistic regression showed that the WHO-FC significantly improved by 0.412 (95% CI: 0.187-0.908, *P* = 0.028). The switch did not show significant improvement in NT-proBNP levels. In addition, the switch was well tolerated.

**Conclusion:**

The switch from bosentan or ambrisentan to macitentan significantly increased the 6MWD in PAH patients, improved the WHO-FC, and exerted safety benefits. The effects of the switch on NT-proBNP levels, hemodynamics, and echocardiography still need to be further confirmed.

**Systematic review registration:**

[https://www.crd.york.ac.uk/prospero/], identifier [CRD42021292554].

## Introduction

The characteristics of pulmonary arterial hypertension (PAH) include high pulmonary vascular resistance, which often results in right heart failure and death (1). Although specific drugs have been developed to treat PAH, the progression of this disease still occurs (2). Currently, some targeted drugs have been approved to treat PAH (3). Specific drug therapies have been used for the treatment of PAH in three key pathophysiological signaling pathways. Prostacyclin analogs and prostacyclin receptor agonists function in the prostacyclin signaling pathway, phosphodiesterase type 5 inhibitors and soluble guanylate cyclase stimulators target the nitric oxide signaling pathway, and endothelin receptor antagonists (ERAs) function in the endothelin signaling pathway (1, 4).

Endothelin receptor antagonists (ERAs) play a role in inhibiting endothelin receptors and thereby blocking the vasoconstrictive effects of factors released by the endothelium, the levels of which are pathologically increased in PAH. As the first available ERA, bosentan is a dual endothelin-A/endothelin-B receptor antagonist. Bosentan has been shown to induce beneficial improvements in the 6-min walk distance (6MWD), World Health Organization functional class (WHO-FC), hemodynamics and time to clinical worsening in PAH of different etiologies (3, 5–7). Over the past couple of years, two additional ERAs, macitentan and ambrisentan, have been approved for the treatment of PAH. The endothelin receptor is responsible for vasoconstriction, and ambrisentan is a selective endothelin-A receptor antagonist (8). In October 2013, macitentan was approved by the Food and Drug Administration (FDA) in the USA. Macitentan is an orally effective drug that blocks dual endothelin receptors, and it was developed by modifying the structure of bosentan, which resulted in increased efficacy and safety. At the same time, it has enhanced tissue penetration and sustained receptor binding (9–12), which allows once-daily dosing compared with bosentan and ambrisentan (9). Thus, macitentan perhaps outperforms either ambrisentan or bosentan for the treatment of PAH. Macitentan has shown improvements in exercise capacity and WHO-FC compared with placebo (13). Moreover, macitentan has been reported to significantly reduce morbidity and mortality even among patients who received macitentan as an additional treatment to establish PAH therapy (14).

Compared with bosentan or ambrisentan, macitentan seems to provide better clinical outcomes. Given these benefits, many patients and clinicians are expected to choose to transition from bosentan or ambrisentan to macitentan therapy (15). However, there are few reports on the clinical experience of converting patients from bosentan or ambrisentan to macitentan. The purpose of our study was to perform a retrospective statistical analysis of observational studies with small sample sizes on the conversion of bosentan or ambrisentan to macitentan to assess the efficacy and safety of this switch in patients with PAH.

## Methods

### Identification strategy

We followed a prespecified protocol (PROSPERO: CRD42021292554) and the standards of the Preferred Reporting Items for Systematic reviews and Meta-Analyses (PRISMA) statement for reporting systematic reviews (Supplementary Table 1) (16). Available literature published before January 2022 was identified from internet databases, including Embase, PubMed, and the Cochrane Library. For the systematic review, the key terms were “bosentan or ambrisentan” and “macitentan” and “pulmonary arterial hypertension.” To ensure that all relevant studies were identified, we also further scrutinized the references of all pertinent articles.

### Inclusion and exclusion criteria

Based on the following inclusion and exclusion criteria, a trial could be selected for inclusion after it passed the assessment. The inclusion criteria were trials in which the participants had a confirmed PAH diagnosis (WHO Group I PH), with no extra limitations on its etiology; trials in which patients were treated with bosentan or ambrisentan and then converted to macitentan; and trials that mentioned at least one measurable comparison outcome. The exclusion criteria were as follows: duplicate studies, meta-analyses, case reports, animal experiments, and studies lacking outcomes of interest.

### Data extraction and endpoints

For each study, we abstracted the following data: the name of the first author, publication year and sample size. The 6MWD, WHO-FC, N-terminal pro-brain natriuretic peptide (NT-proBNP) levels, hemodynamics and echocardiography were used as endpoints. Data on survival and adverse events were also collected.

### Methodological quality

Two reviewers (JL and Z-YY) used the Newcastle–Ottawa Scale (NOS) to evaluate the methodological quality of the included cohort studies. This scale rates studies on three major domains: selection, comparability, and exposure/outcome. A study with a score of 7 or higher was considered high-quality. Moderate-quality studies were scored between 4 and 6, and low-quality studies had a score less than 4 (17).

### Statistical analysis methods

We used Review Manager (RevMan) [computer program] [Version 5.4, The Cochrane Collaboration] to perform data management, transformation of effect size, and calculation of pooled prevalence. The inverse variance method and a random-effects model were performed for the meta-analyses of dichotomous outcomes. We computed fixed- and random-effects models but considered only the random-effects model when heterogeneity was detected. To assess the heterogeneity between studies, the Q-statistics were calculated, where *P* < 0.10 was considered statistically significant. The percent of observed variation across studies caused by heterogeneity was estimated by calculating *I*^2^. *I*^2^ ranged from 0 to 100%, and there were three categories of *I*^2^ values: low (<25%), moderate (25%–50%), and high (50%–75%). Ordinal logistic regression models included fixed effects to test whether conversion from bosentan/ambrisentan to macitentan affected the WHO-FC outcome. When more than 10 studies were included in the meta-analysis, we used funnel plot asymmetry to detect publication bias (18).

## Results

### Characteristics of the studies

**FIGURE 1:**
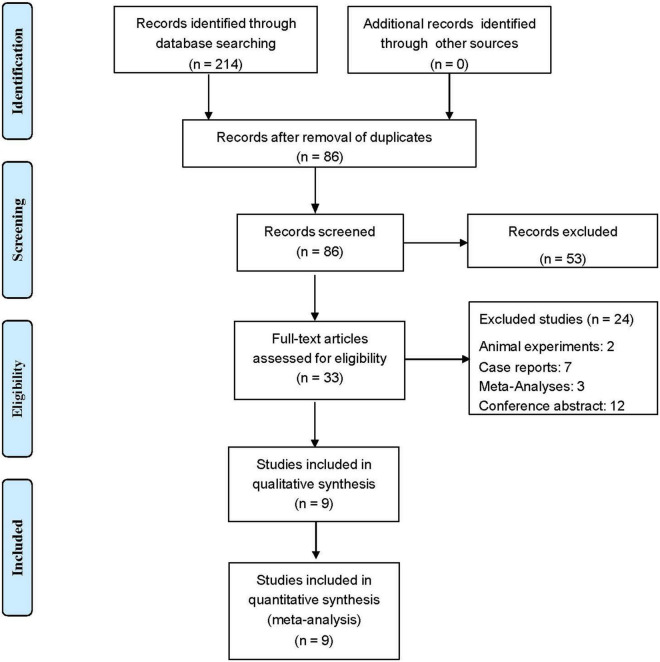
The PRISMA flowchart.

Figure 1 shows the flow diagram used for selection. For the initial systematic search, 214 results were retrieved, and 86 results remained after deleting duplicate results. The preprint platform returned 10 records. Of the identified records, 33 articles were thought to have high potential to meet the inclusion criteria. After reading the full text of each article and carefully screening it, we excluded 24 articles (12 conference abstracts, 7 case reports, 3 meta-analyses and 2 animal experiments). Finally, we identified 9 eligible articles. Table 1 summarizes the study characteristics and quality assessments of all included articles.

**TABLE 1:** Characteristics of included studies.

References	Number/Age (years)/Female (%)/Design type	Follow-up time	Conversion	Main outcome measures	Results
Tynan et al. (24)	⋅ 37 ⋅ 63 ± 13 ⋅ 12 (85.7) ⋅ CS (retrospective)	18 months	B/A → M	6MWD, WHO-FC, Echocardiography, Safety, etc.	–: 6MWD, RVS, PASP, etc.
Aypar et al. (20)	**⋅** 13 **⋅** 20.3 ± 6.5 **⋅** 8 (61.5) **⋅** CS (prospective)	24 weeks	B → M	6MWD, WHO-FC, BNP, SaO_2_, Echocardiography, Hemodynamics, etc.	↑: 6MWD, etc. –: SaO2, BNP, sPAP, etc.
Schweintzger et al. (25)	**⋅** 18 **⋅** 8.5 (0.6, 16.8) **⋅** 8 (44.4) **⋅** CS (prospective)	6 months	B → M	6MWD, WHO-FC, NT-proBNP, Echocardiography, Hemodynamics, etc.	↑: TAPSE, NT-proBNP, mPAP/mSAP, PVRi, etc. –: 6MWD, WHO-FC, etc.
Bouma et al. (21)	**⋅** 40 **⋅** 45 ± 13 **⋅** 16 (40.0) **⋅** CS (prospective)	6 months	B → M	6MWD, WHO-FC, NT-proBNP, SaO_2_, Echocardiography, etc.	↑: WHO-FC, NT-proBNP, TAPSE, etc. –: 6MWD, SaO_2_, etc.
Safdar et al. (23)	**⋅** 24 **⋅** 58 ± 13 **⋅** 21 (87.5) **⋅** CS (retrospective)	6 months	B → M	6MWD, WHO-FC, BNP, Echocardiography, Hemodynamics, Safety, etc.	–: 6MWD, BNP, CO, CI, AST, ALT, etc.
Cadenas-Menéndez et al. (22)	**⋅** 12 **⋅** 65.63 ± 13.27 **⋅** 10 (83.3) **⋅** CS (retrospective)	12 months	B/A → M	6MWD, WHO-FC, NT-proBNP, Echocardiography, etc.	↑: 6MWD, etc. –: NT-proBNP, WHO-FC, etc.
Dawson et al. (26)	**⋅** 92 **⋅** 58 ± 14 **⋅** 36 (73) **⋅** CS (retrospective)	43 months	B → M	6MWD, NT-proBNP, Hemodynamics, Safety, etc.	↑: NT-proBNP, CI, mPAP, RAP, etc.
Aypar et al. (19)	**⋅** 27 **⋅** 21.1 ± 6.3 **⋅** 20 (74.1) **⋅** CS (prospective)	22 months	B → M	6MWD, WHO-FC, Safety, etc.	↑: 6MWD, etc.
Chen et al. (15)	**⋅** 145 **⋅** 32.0 (26.0, 42.0) **⋅** 108 (74.5) **⋅** CS (prospective)	12 months	B → M	6MWD, WHO-FC, Hemodynamics, Safety, etc.	↑: 6MWD, NT-proBNP, TAPSE, RVFAC, sPAP, etc. –: LVEDD, etc.

↑ represents a significant improvement in the measures compared to those before macitentan initiation. “–” represents no significant improvement in the measure compared to those before macitentan initiation. 6MWD, 6-min walking distances; A, ambrisentan; ALT, alanine transaminase; AST, aspartate transaminase; B, bosentan; BNP, brain natriuretic peptide; CI, cardiac index; CO, cardiac output; CS, cohort Study; LVEDD, left ventricular end-diastolic dimension; M, macitentan; mPAP, mean pulmonary arterial pressure; mPAP/mSAP, the ratio of mean pulmonary arterial pressure/mean systemic arterial pressure; NT-pro BNP, N-terminal pro-brain natriuretic peptide; PAP, pulmonary arterial pressure; PASP, pulmonary artery systolic pressure; PVRi, pulmonary vascular resistance index; RAP, right atrial pressure; RVFAC, right ventricular area change rate; SaO2, arterial oxygen saturation; sPAP, systolic pulmonary artery pressure; TAPSE, tricuspid annular plane systolic excursion; WHO-FC, World Health Organization functional class.

### Quality assessment

All 9 articles were cohort studies, and all articles were assessed for methodological quality. According to the NOS assessment, six studies were rated as high quality. Three studies were rated as moderate quality (Supplementary Table 2).

### Effectiveness

#### Six-minute walking distance

**FIGURE 2:**
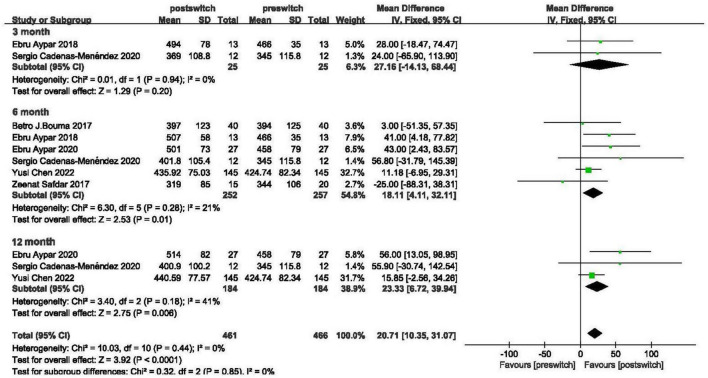
Changes in the 6-min walk distance after transition.

Exercise capacity was assessed with 6MWD changes in 6 studies (15, 19–23). Biased studies were excluded, and a fixed-effects model was applied, as shown in Figure 2, which showed an increase in the 6MWD of 20.71 m (95% CI: 10.35–31.07, *P* < 0.00001, *I*^2^ = 0%). In the subgroup analysis, the 6MWD improved by 27.16 m, 18.11 m and 23.33 m at 3 months, 6 months and 12 months after conversion with mild heterogeneity (95% CI: −14.13 to 68.44 m, *P* = 0.20, *I*^2^ = 0%; 95% CI: 4.11–32.11 m, *P* = 0.01, *I*^2^ = 21%; 95% CI: 6.72–39.94 m, *P* = 0.006, *I*^2^ = 41%, respectively).

#### World Health Organization functional class

In 3 studies (22–24), ordinal logistic regression showed that the WHO-FC significantly improved by 0.412 (95% CI = 0.187–0.908, *P* = 0.028) [number of observations = 96 LR chi^2^(1) = 4.96, Prob > chi^2^ = 0.0259, Log likelihood = −94.118619, Pseudo *R*^2^ = 0.0257].

#### N-terminal pro-brain natriuretic peptide

**FIGURE 3:**
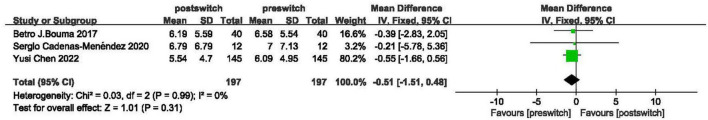
Changes in N-terminal pro-brain-type natriuretic peptide levels after transition.

Three studies reported NT-proBNP levels in adults (15, 21, 22). After logarithmic transformation, NT-proBNP levels did not show significant improvement (MD = -0.51, 95% CI: −1.51 to 0.48, *P* = 0.31, *I*^2^ = 0%). In addition, Schweintzger et al. found that NT-proBNP levels decreased from 6.47 ± 6.53 to 5.78 ± 5.09 pg/ml in children (25) (Figure 3).

#### Hemodynamic parameters

Six months after conversion, Dawson et al. found that the mean pulmonary arterial pressure (mPAP) decreased from 51.2 ± 9.0 to 47.0 ± 12.2 mmHg and that the right atrial pressure (RAP) decreased from 10.7 ± 4.2 to 9.0 ± 3.9 mmHg in adults (26). Safdar et al. found that the cardiac index (CI) decreased from 3.4 ± 4.2 L/(min⋅m^2^) to 3.3 ± 3.3 L/(min⋅m^2^) in adults (23). For children, 6 months after conversion, Schweintzger et al. found that the CI increased from 3.35 ± 0.8 to 3.85 ± 1.3 L/(min⋅m^2^) (25). At the same time, Schweintzger et al. also found that mPAP increased from 37.5 ± 24.0 to 38.0 ± 15.0 mmHg and that RAP increased from 9.0 ± 6.0 to 10.0 ± 5.0 mmHg in children over 6 months after conversion.

### Echocardiography

Six months after conversion, Bouma et al. found that the tricuspid annular plane systolic excursion (TAPSE) improved from 19.0 ± 4.0 to 21.0 + 5.0 mm in adults (21). Schweintzger et al. found that TAPSE improved from 16.00 ± 5.0 to 18.25 ± 4.8 mm in children (25).

### Survival analysis

Of all included studies, only the study by Dawson et al. described survival (26). However, in addition to patients who transitioned from bosentan to macitentan (*n* = 49), the population in their study also included those who transitioned from bosentan to ambrisentan (*n* = 43). In the total population, a median follow-up time of 20.08 (IQR, 12.37–25.98) months elapsed between transition to death or the time of last review. According to the Kaplan–Meier estimation, the survival rates at 1 and 2 years were 93.1% and 81.3% after transition, respectively. Seventeen patients died during the entire study period. Of these, 5 patients transitioned from bosentan to macitentan, and 12 patients transitioned from bosentan to ambrisentan.

### Safety

Supplementary Table 3 compares the adverse events (AEs) that occurred in the articles that were included.

Adverse events (AEs) that occurred during the 6 months after drug conversion were counted (15, 24, 26). The most common AEs were peripheral edema (20.0%), anemia (19.5%), ankle edema (15.2%), headache (14.7%), liver dysfunction (14.2%) and menstrual disorder (11.4%). Overall, few patients ended up discontinuing macitentan due to AEs. In addition, according to Chen et al. (15), we found that anemia and female menstrual irregularities in females were significantly higher after transition than before transition and that females of reproductive age mostly suffered severe anemia.

## Discussion

Our study showed that as a novel ERA, macitentan exhibits beneficial effects in PAH patients; the switch from other ERAs (bosentan or ambrisentan) to macitentan can improve the 6MWD and WHO-FC and have safety benefits.

At present, bosentan, ambrisentan and macitentan are all approved ERA medications for PAH. Bosentan is a dual antagonist of endothelin receptor A and B with a half-life of 5 h. However, it may increase the incidence of dose-dependent increases in liver aminotransferase concentrations (27). In addition, ambrisentan is the only available selective ERA (ET-A receptor antagonist) with a half-life of 15 h. Studies have shown that the combination of ambrisentan with tadalafil could decrease the risk of PAH-related hospitalization compared with monotherapy and has a benefit of delayed clinical deterioration in PAH (28, 29). However, there is still a lack of large-scale studies that focus on the improvement in long-term morbidity and mortality with ambrisentan monotherapy. Macitentan is an orally active, potent, dual endothelin receptor antagonist developed by modifying the structure of bosentan. Macitentan has slower receptor dissociation kinetics and a longer duration of action, which allows once-daily dosing and freedom from monthly liver function tests (30). Moreover, a study demonstrated that macitentan achieved a greater reduction in mPAP than bosentan (21). Macitentan also appears to be superior with respect to hepatic safety and edema than bosentan and ambrisentan, respectively. Therefore, macitentan might be an alternative or even superior to the other two ERAs.

The clinical efficacy of macitentan, as a new-generation drug, has been confirmed by many studies. The SERAPHIN study showed that macitentan can improve exercise ability, clinical symptoms and outcomes, including mortality, hospitalization rate, WHO-FC, and 6MWD (13). The REPAIR study showed that macitentan is beneficial to right ventricular (RV) structure and function in PAH (31). Our analysis included parameters used for simple risk stratification of PAH (32), including the WHO-FC, 6MWD, NT-proBNP levels, RAP and the CI. This study showed that the switch from bosentan or ambrisentan to macitentan can improve two parameters that compose the risk scores, namely, the 6MWD and WHO-FC. The 6MWD is a parameter that is used to evaluate exercise capacity. The WHO-FC is a robust measure that has been used to guide therapy. These findings confirmed that the transition to macitentan is effective for the treatment of PAH. Nevertheless, as the number of studies on RV function is limited, we could not analyze the RV structure and function by cardiac magnetic resonance as the REPAIR study described. The sample size included in the study was relatively small, so we could not pool the CI analysis, which reflects cardiac function in hemodynamics. However, the TAPSE, another longitudinal parameter of RV contraction potential detected by echocardiography, was improved in both children and adults, which indicated that conversion also improved right heart function to some extent. The reasons for the improvement accompanying the transition may be as follows: first, the transition from ambrisentan to macitentan optimized the drug exposure by means of eliminating the drug interaction of ambrisentan, and second, the dual endothelin receptor antagonist may have superiority.

In China, Chen et al. conducted a real-world prospective study and followed the participants for 12 months (15). PAH patients were treated with stable doses of ambrisentan for over 3 months. Then, after a transition phase, patients with PAH would undergo long-term follow-up. The results of Chen et al. showed that the transition could improve the exercise capacity, cardiac function, and hemodynamics, namely, the 6MWD, WHO-FC, NT-proBNP levels, quality of life, REVEAL Risk scores and right heart function, compared with those at baseline. In this study, significant improvements in NT-proBNP levels were not observed. The difference between our results and the results of Chen et al. may be attributed to the very small number of studies included.

Compared with placebo, macitentan showed benefits in the amelioration of clinical worsening in PAH patients and reduced the hospitalization rates related to PAH in the SERAPHIN study. Macitentan is the first ERA has provides tissue specificity. It has demonstrated efficacy for morbidity and mortality in patients with PAH. However, of all included studies, the study by Dawson et al. involved the survival of patients switching from other ERAs to macitentan (26). Although 5 patients who transitioned from bosentan to macitentan died, and none of the deaths occurred as a result of ERA treatment. Transitioning from bosentan to macitentan was well tolerated, and there were no reports about safety concerns related to the transition itself. However, given that the study by Dawson et al. included both conversion from bosentan to macitentan and conversion from bosentan to ambrisentan, we may need to carry out further conversions of bosentan to macitentan only to better understand the safety of conversion from other ERAs to macitentan.

In terms of safety, some studies have reported that hepatotoxicity, peripheral edema, and anemia are associated with traditional ERAs but not with macitentan. In our analysis, we found that few patients discontinued macitentan due to AEs, which showed that there was a good tolerability and safety profile for this transition. Dawson et al. (26) thought that all AEs were possibly in accordance with those reported in clinical trials, and neither of the deaths was attributed to the transition protocol itself. Chen et al. (15) found that although the incidence of AEs was high, most AEs were mild to moderate. In addition, unlike the majority of AEs that exhibited similar characteristics before and after drug conversion, the proportion of anemia and menstrual disorders increased 6 months after conversion. At the same time, anemia may be associated with menstrual disorders in some patients. Therefore, anemia and menstrual disorders in female patients need to be carefully considered. This suggests that in clinical practice, we should pay special attention to women undergoing transition therapy who present with anemia and menstrual disorders.

### Limitations

First, the number of studies that have assessed the efficacy and safety of the transition from bosentan/ambrisentan to macitentan among PAH patients was limited, which affected the reliability of our conclusions to some extent. In addition, the sample size of the included studies was small. Although there were several randomized controlled trials (RCTs) that assessed the efficacy of the transition from bosentan or ambrisentan to macitentan, the data were not available. Second, most of the included studies were from single centers, and there is a need for multicenter RCTs to better characterize the long-term benefit of the transition from bosentan or ambrisentan to macitentan in the future. Third, there are no data regarding long-term survival after switching to macitentan. Finally, we could not completely eliminate the selection bias in any meta-analyses.

## Conclusion

The present meta-analysis findings indicate that the switch from bosentan or ambrisentan to macitentan may improve the 6MWD and WHO-FC, which may provide evidence for the clinical treatment of PAH and have certain importance in the study of cardiopulmonary vascular diseases. However, more large-scale, multicenter studies are needed to confirm the effectiveness and safety of this switch in patients with PAH.

## Data availability statement

The raw data supporting the conclusions of this article will be made available by the authors, without undue reservation.

## Author contributions

RJ and S-SL contributed to the conception and design of the study. JL, Z-YY, and SW organized the database. H-LQ, C-JL, and Q-HZ performed the statistical analysis. PY, RJ, and SW were responsible for the first draft of the manuscript. S-GG, J-ML, RZ, H-TL, W-HW, and LW wrote sections of the manuscript. All authors were involved in revisions to the manuscript and have read and approved its submission.
