# Supplementary material for: Efficacy and safety of switching from bosentan or ambrisentan to macitentan in pulmonary arterial hypertension: A systematic review and meta-analysis
Source: Front Cardiovasc Med. 2022 Dec 7;9:977110. doi: 10.3389/fcvm.2022.977110 (PMC9767980; doi:10.3389/fcvm.2022.977110)
Supplement: Supplementary file 3 [file Table_3.DOCX]

| **e-Table 3 Adverse events in patients who transitioned to macitentan (6 months).** | | | |
| --- | --- | --- | --- |
| **Adverse events** | **Total** | **+** | **Percentage(%)** |
| Ankle oedema | 46 | 7 | 15.2 |
| Headache | 191 | 28 | 14.7 |
| Muscle cramps | 46 | 1 | 2.2 |
| Joint ache | 46 | 1 | 2.2 |
| Cold symptoms/nasal stuffiness | 46 | 2 | 4.4 |
| Stomachache | 46 | 1 | 2.2 |
| Nausea/vomiting | 46 | 2 | 4.4 |
| Anemia | 159 | 31 | 19.5 |
| Liver derangement | 14 | 2 | 14.2 |
| Peripheral edema | 145 | 29 | 20.0 |
| Menstrual disorder | 79 | 9 | 11.4 |

**Note:** “+” represents the number of people with adverse events.
